# Challenges in Providing an Overview of Results of Intermittent Fasting Interventions on Diabetes Parameters. Comment on Silva et al. Effects of Intermittent Fasting on Regulation of Metabolic Homeostasis: A Systematic Review and Meta-Analysis in Health and Metabolic-Related Disorders. *J. Clin. Med.* 2023, *12*, 3699

**DOI:** 10.3390/jcm13144091

**Published:** 2024-07-12

**Authors:** Carmen Dietvorst, Jur Kroon, Romy Slebe, Mireille J. Serlie, Kirsten A. Berk, Femke Rutters

**Affiliations:** 1 Department of Internal Medicine, Division of Dietetics, Erasmus Medical Centre, 3015 GD Rotterdam, The Netherlands; c.dietvorst@erasmusmc.nl (C.D.);; 2 Department of Endocrinology and Metabolism and Amsterdam Gastroenterology Metabolism Endocrinology Institute, Amsterdam University Medical Center, 1081 BT Amsterdam, The Netherlands; jur.kroon@amsterdamumc.nl (J.K.);; 3 Department of Epidemiology and Data Science, Amsterdam University Medical Centre, 1105 AZ Amsterdam, The Netherlands; 4 Section of Endocrinology, Department of Internal Medicine, Yale School of Medicine, New Haven, CT 06510, USA

We hereby comment on the systematic review “Effects of Intermittent Fasting on Regulation of Metabolic Homeostasis: A Systematic Review and Meta-Analysis in Health and Metabolic-Related Disorders” by Silva et al., 2023 [1]. We appreciate the herculean effort undertaken to summarize the extended research on effects of intermittent fasting on several metabolic parameters. Intermittent fasting was defined by Silva et al., 2023 as Alternate-Day Fasting, Time-Restricted Fasting a.k.a. time-restricted eating, fasting-mimicking diet as well as Religious Fasting. However, there are also some concerns. While performing an umbrella review on the topic of intermittent fasting and (parameters of) diabetes, we encountered the following problems; first, there is a large amount of available reviews on a seemingly small amount of original papers. Within the same timeframe, we identified 116 original studies on intermittent fasting and diabetes, compared to 51 reviews. Table 1 provides an overview of all the reviews identified to date for our umbrella review and the % inclusion of the original studies.

The systematic review by Silva et al. [1] included only 30% of available original studies, which is more than the other reviews, which included on average 10%. We think the review of Silva et al., 2023 [1] is incomplete due to:

- *The different forms and definitions of intermittent fasting*: intermittent fasting can refer to Time-Restricted Fasting/Time-Restricted Eating, Alternate-Day Fasting, Modified Alternate-Day Fasting regimes, Fasting-Mimicking diets as well as fasting for religious reasons. In the Silva et al. [1] review Fasting-Mimicking diets were not included;
- *A lack of alternative search strategies*, such as checking references. We were surprised that the authors identified no papers through manual searching [52], while about one-fourth of the included papers in our umbrella review were identified through reference checking;
- *Selection of outcomes*: the review of Silva et al. included a variety of metabolic outcomes, but, for diabetes parameters only insulin homeostasis, fasting glucose, fasting insulin, insulin resistance (HOMA-IR) and diabetes status were considered. However, there are other important diabetes parameters to be considered when studying the effects of intermittent fasting. These include HbA1c, OGTT (1 h and 2 h post-load glucose), Time in Range, glucose variation, hyperglycemia and hypoglycemia, postprandial glucose, insulin responses, C-reactive protein, glucagon, other insulin resistance indexes (ISI, OGIS, MCR, Matsuda) and hyperglycemic as well as euglycemic clamps to assess beta-cell function and insulin sensitivity respectively;
- *The exclusion criteria*, excluding studies that combined IF with other diets or interventions, which presumably led to a selection of studies.

The other reviews summarized in Table I have more missing studies, compared to Silva et al. [1]. This might be caused by the reasons outlined above and by the strict inclusion and exclusion criteria applied in some of those reviews. For example only including studies in specific subgroups like women with PCOS [51] or only studies on Ramadan fasting [39], which limits the generalizability of those reviews.

Although, the systematic review by Silva et al., 2023 [1] adds valuable information to the literature on the effects of intermittent fasting on metabolic parameters, we feel that a complete review, which includes all original studies on the topic of intermittent fasting and diabetes is still missing from the literature. The lack of such a complete systematic overview on the effect of all forms of intermittent fasting on all relevant diabetes related outcomes and with emphasis on a comprehensive evidence synthesis of the outcomes per type of intermittent fasting refrains researchers and health care professionals from implementing the best intermittent fasting diet for people with diabetes. 

## Figures and Tables

**Table 1:** Overview of identified (systematic) reviews on intermittent fasting and per review included single studies, ordered from highest number of original studies included to lowest.

Table of Reviews			
Author	Review Type	Number of Studies Included	Percentage of Total
Silva et al., 2023 [1]	Systematic review and meta-analysis	35	30.17%
Yang et al., 2021 [2]	Systematic review	33	28.45%
Gu et al., 2022 [3]	Systematic review and meta-analysis	30	25.86%
Sharma et al., 2023 [4]	Systematic review and meta-analysis	21	18.10%
Tsitsou et al., 2022 [5]	Systematic review	20	17.24%
Allaf et al., 2021 [6]	Systematic review and meta-analysis	19	16.38%
Liu et al., 2023 [7]	Systematic review and meta-analysis	19	16.38%
Wang et al., 2022 [8]	Systematic review and meta-analysis	19	16.38%
Sun et al., 2023 [9]	Systematic review and meta-analysis	19	16.38%
de Oliveira Maranhão Pureza 2021 [10]	Systematic review and meta-analysis	18	15.52%
Chen et al., 2023 [11]	Systematic review and meta-analysis	18	15.52%
Parr et al., 2022 [12]	Perspective	18	15.52%
Kyoung-Kon Kim et al., 2022 [13]	Systematic review and meta-analysis	17	14.66%
Chen et al., 2021 [14]	Systematic review and meta-analysis	17	14.66%
van den Burg et al., 2023 [15]	Systematic review and meta-analysis	17	14.66%
He et al., 2020 [16]	Systematic review and meta-analysis	15	12.93%
Ezzati et al., 2023 [17]	Systematic review	15	12.93%
Moon et al., 2020 [18]	Systematic review and meta-analysis	14	12.07%
Liu et al., 2022 [19]	Systematic review and meta-analysis	14	12.07%
Borgundvaag 2021 [20]	Systematic review and meta-analysis	13	11.21%
Cioffi et al., 2018 [21]	Systematic review and meta-analysis	12	10.34%
Schwingshackl et al., 2021 [22]	Systematic review and meta-analysis	12	10.34%
Cho et al., 2019 [23]	Systematic review and meta-analysis	12	10.34%
Meng et al., 2020 [24]	Systematic review and meta-analysis	12	10.34%
Fatahi et al., 2021 [25]	Systematic review and meta-analysis	12	10.34%
Wang et al., 2021 [26]	Systematic review and meta-analysis	11	9.48%
Nowosad et al., 2021 [27]	Systematic review	11	9.48%
Ciu et al., 2020 [28]	Systematic review and meta-analysis	9	7.76%
Santos et al., 2022 [29]	Scoping review	9	7.76%
Kirkham et al., 2022 [30]	Narrative review	9	7.76%
Bórquez et al., 2022 [31]	Narrative review	9	7.76%
Navea-Cuadra et al., 2022 [32]	Systematic review	8	2.59%
Yuan et al., 2022 [33]	Systematic review and meta-analysis	8	6.90%
Park et al., 2020 [34]	Systematic review and meta-analysis	7	6.03%
Roman et al., 2018 [35]	Systematic review and meta-analysis	7	6.03%
Barnosky et al., 2014 [36]	Systematic review	7	6.03%
Lin et al., 2022 [37]	Systematic review	7	6.03%
Morales-Suarez-Varela et al., 2021 [38]	Systematic review	7	6.03%
Pellegrini et al., 2020 [39]	Systematic review and meta-analysis	6	5.17%
Anton et al., 2021 [40]	Systematic review	6	5.17%
Zaki et al., 2022 [41]	Systematic review and meta-analysis	6	5.17%
Harris et al., 2018 [42]	Systematic review and meta-analysis	5	4.31%
Chew et al., 2023 [43]	Systematic review and meta-analysis	5	4.31%
Zeng et al., 2022 [44]	Systematic review and meta-analysis	5	4.31%
Vitale et al., 2020 [45]	Systematic review	5	4.31%
Bitsanis et al., 2021 [46]	Systematic review	5	4.31%
Ganesan et al., 2020 [47]	Systematic review	5	4.31%
Osses-Carrasco et al., 2022 [48]	Systematic review	4	3.45%
Uldal et al., 2022 [49]	Systematic review	4	3.45%
Patikorn et al., 2021 [50]	Umbrella review	2	1.72%
Floyd et al., 2022 [51]	Systematic review	1	0.86%
